# Association between pregnancy loss and depressive symptoms in women: a population-based study

**DOI:** 10.1186/s12888-024-05948-0

**Published:** 2024-07-23

**Authors:** Shan Wang, Yuan Wang, Ling Tong, Jiaru Zhuang, Dewu Xu, Yibo Wu

**Affiliations:** 1 Obstetrics, Gynecology and Reproduction Research, Affiliated Hospital of Jiangnan University, 1000 Hefeng Road, Wuxi, Jiangsu 214000 P.R. China; 2 Department of Medical Education, Affiliated Hospital of Jiangnan University, 1000 Hefeng Road, Wuxi, Jiangsu 214000 P.R. China

**Keywords:** Depression, Pregnancy loss, NHANES, A cross-sectional study

## Abstract

**Background:**

Depression is a common mental disorder with a much higher prevalence in women than in men. Although there has been a gradual increase in research on the association between reproductive health and depression, there is still some inconsistency in the evidence of the relationship between pregnancy loss and depression. This study aimed to investigate the relationship between pregnancy loss and depressive symptoms.

**Methods:**

We analyzed data from the 2007–2020 National Health and Nutrition Examination Survey (NHANES), which included female participants aged 20 to 80 years. Pregnancy loss was determined based on participants’ self-reported number of pregnancies and pregnancy outcomes. Depressive symptoms were measured using the Patient Health Questionnaire (PHQ-9, score ≥ 10). Multivariate logistic regression, smoothed curve fitting, and generalized additive modeling were used to examine the association between pregnancy loss and depression. We also performed sensitivity analyses and subgroup analyses to verify the robustness and specificity of the findings.

**Results:**

A total of 12,873 female participants were included in our study, of which 1,595 (12.39%) were categorized as depressed. Multivariate logistic regression results indicated that experiencing a pregnancy loss increased the risk of prevalence of depression in women (for 1 loss: OR = 1.31, 95% CI 1.15,1.50; for 2 or more losses: OR = 1.58, 95% CI 1.38, 1.81). When sensitivity analyses were performed, an association between pregnancy loss and depression was found in both multivariate linear regressions with PHQ-9 scores as a continuous variable and multivariate logistic regressions with a threshold of 5 PHQ-9 scores. The association between pregnancy loss and depression remained stable across subgroups.

**Conclusion:**

Pregnancy loss correlated with elevated PHQ-9 scores and a heightened risk of depression in adult women across the United States. Focusing on the incidence of adverse pregnancy events in the female population may help prevent or early recognize the onset of depression.

## Highlights

We analyzed the relationship between pregnancy loss and depression in American adult women. The sample size was large and nationally representative.

Pregnancy loss was associated with higher PHQ-9 scores and an increased likelihood of depression among adult women.

Our study is expected to provide more effective strategies to mitigate the impact of adverse pregnancy events on women’s mental health.

## Introduction

Major Depressive Disorder (MDD), also known as depression, significantly contributes to the global health burden, impacting individuals’ emotions, cognition, and behaviors [1]. Unlike fleeting sadness or temporary mood dips, this disorder is marked by continuous despondency, disinterest, and reduced ability, potentially escalating to suicide in its most extreme form [2]. The Global Burden of Disease (GBD) study from 1990 to 2017 shows a generalized upward trend in the prevalence of depression [3]. It is widely acknowledged that depression’s roots are complex and multifaceted, involving genetic, biochemical, environmental, and psychosocial elements. From the lens of biomedical science, an imbalance in brain neurotransmitters such as serotonin [4], norepinephrine [5], and dopamine [6] is closely linked to depression. Additionally, alterations in neuroendocrinology, especially the dysregulation of cortisol, a stress hormone, are crucial in understanding depression’s onset [7]. Notably, there exists a disparity in depression incidence between genders, with it being nearly twice as prevalent in females as in males, as per numerous studies [8].

Both spontaneous pregnancy losses, such as miscarriages or stillbirths, and elective pregnancy losses, such as induced abortions, cause profound suffering to the mother. These losses during pregnancy have a serious impact on maternal mental health issues, while increasing the risk of suicidal behavior [9]. Acknowledged by the World Health Organization as a critical public health concern, miscarriages affect about 15–20% of acknowledged pregnancies, and stillbirths occur in approximately 1 in 160 pregnancies every year across the globe [10]. The consequences of abortion are not only physical but also deeply affect mental health, as it is a painful process [11]. In contrast, women with severe mental disorders (e.g., anxiety) have significantly higher rates of abortion, especially spontaneous abortion [12]. The profound sorrow and trauma stemming from pregnancy loss can impact not only the health of the woman, but also that of her partner, children, and broader family [13]. The emergence of common mental health disorders (CMHD) such as anxiety, depression, and post-traumatic symptoms (PTS) following such an event is believed to be driven by a complex interaction of biological shifts, individual susceptibilities to mental health disorders, and societal stigma and isolation often linked to pregnancy loss [14, 15].

Based on the above understanding, it is necessary to explore whether there is an association between pregnancy loss and depression. Addressing this concern is vital for enhancing mental health outcomes and offering holistic care to women confronted with the profound effects of pregnancy loss. Hence, this study aimed to investigate the association between pregnancy loss and depressive symptoms among adult women in the United States, utilizing data from the 2007–2020 National Health and Nutrition Examination Survey (NHANES). This extensive cross-sectional analysis endeavors to furnish sound theoretical support for understanding the occurrence of depressive symptoms following pregnancy loss in women.

## Materials and methods

### Study population and design

**Fig. 1:**
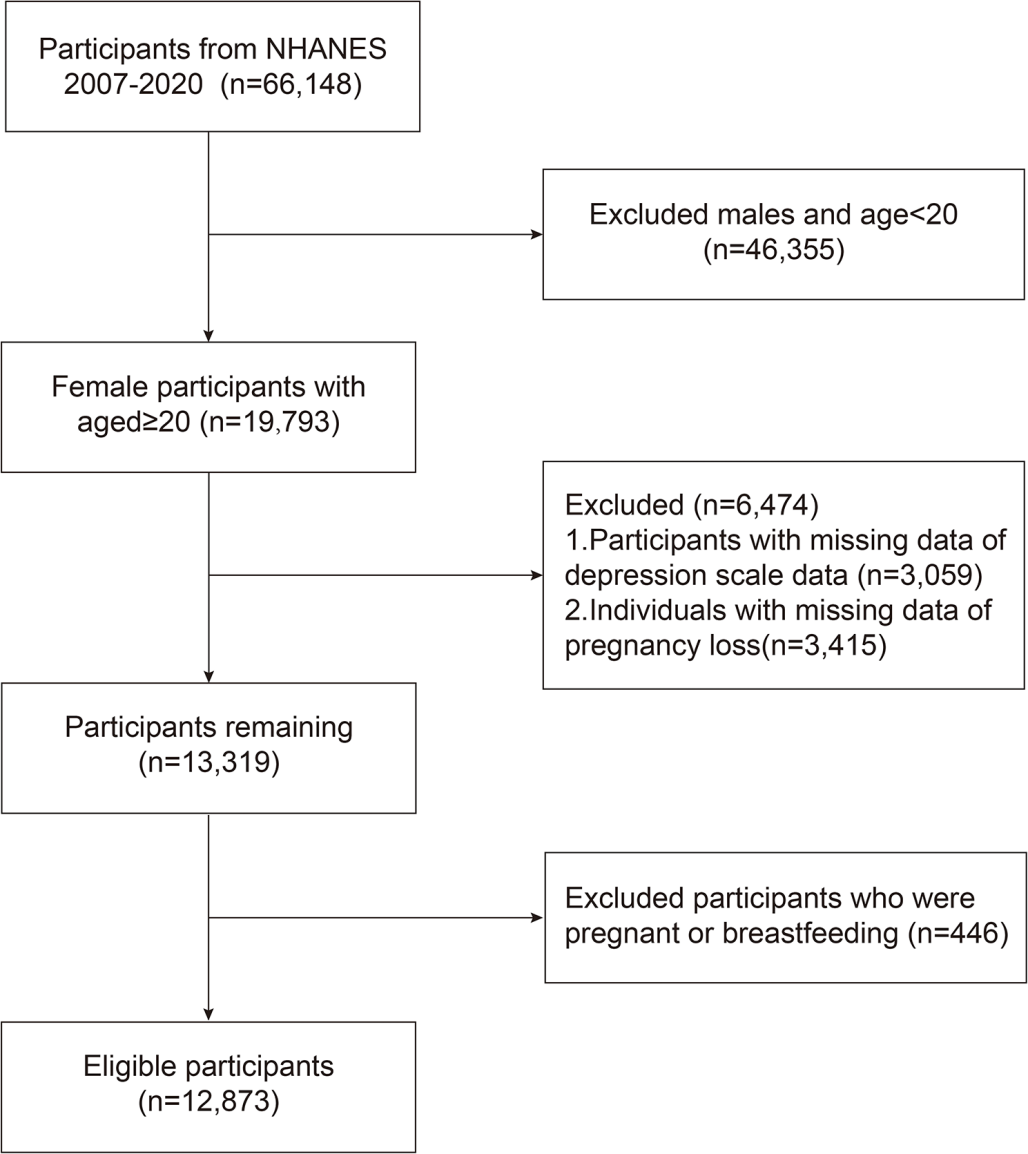
Flow chart of participants selection

For this investigation, we harnessed data from the National Health and Nutrition Examination Survey (NHANES), an extensive dataset encompassing demographic, dietary, physical, laboratory, and imaging information to assess the health and nutritional status of a representative sample of the US population [16]. NHANES employs a sophisticated, stratified, multistage probability sampling methodology, yielding a sample of approximately 10,000 noninstitutionalized civilians annually. Selected individuals are scheduled for a telephone health interview and a face-to-face interview by NHANES staff. Following the interview, the participant is scheduled for a physical examination at the Mobile Exam Center (MEC). All of the above-standardized interviews and physical examinations are completed at one point in time. Participants from 2007 to 2020 were included in this study due to the availability of comprehensive reproductive-related data. The research protocol secured approval from the National Center for Health Statistics (NCHS) Research Ethics Review Board. Furthermore, every participant engaged in the study provided informed written consent, ensuring adherence to ethical research standards. Initially, 66,148 participants from the specified NHANES cycles were identified. We excluded 32,793 male participants, 13,562 participants younger than 20 years of age, 3,059 participants with missing data on the PHQ-9 questionnaire, and 3,415 participants with missing data on pregnancy outcomes. Because the NHANES did not collect complete information from participants 18–19 years, the lower age of inclusion in this study was 20 years instead of 18. In addition, we excluded 446 participants who were pregnant or breastfeeding to eliminate the possibility that women reported increased depressive symptoms due to fluctuating hormone levels before pregnancy and during breastfeeding. Finally, a total of 12,873 women participated in the study (Fig. 1).

### Pregnancy loss

Pregnancy loss refers to the termination of a pregnancy at any stage and primarily involves miscarriages, stillbirths, and elective terminations [17]. Pregnancy loss was assessed based on self-reported outcomes of reproductive health from computer-assisted personal interviews conducted at the Mobile Examination Center (MEC). Participants were asked two questions: (1) How many times have you been pregnant? Be sure to count all your pregnancies including current pregnancy, live births, miscarriages, stillbirths, tubal pregnancies, and other pregnancies. (2) How many of your deliveries resulted in a live birth? The criterion for recognizing a pregnancy loss was when the tally of reported pregnancies surpassed the count of live births by at least one in individuals not currently pregnant. The study further divided instances of pregnancy loss into three distinct categories for analysis: none, one, and two or more.

### Depression

From 2007 to 2020, the assessment of depressive symptoms in participants employed the Depression Questionnaire PHQ-9 (Patient Health Questionnaire-9), a diagnostic tool for initial screening and evaluation of depression severity [18]. It aligns with the major depressive disorder criteria from the Diagnostic and Statistical Manual of Mental Disorders, Fourth Edition, scoring responses based on symptom frequency over the preceding two weeks on a scale from 0 (not at all) to 3 (nearly every day). Thus, total scores could range from 0 to 27, with higher scores denoting more acute depressive symptoms. This study adopted a PHQ-9 score of 10 or above as indicative of “yes” to depressive symptoms, leveraging a threshold previously established as having 88% sensitivity and specificity. In sensitivity analyses, we also used a threshold score of 5 to define mild depressive symptoms or more, with a total PHQ-9 score of < 5 indicating no depression and ≥ 5 indicating mild depression.

### Covariates

The selection of covariates was informed by clinical insights and prior research findings. Based on the availability of NHANES data, the following covariates of interest were included in this study: (a) Demographic and socioeconomic information, including age, age at menarche, age at first delivery, age at last delivery, the number of pregnancies, the number of live births, the number of vaginal deliveries, the number of cesarean deliveries, race (categorized as non-Hispanic white, non-Hispanic black, Mexican American, other Hispanic or other races), education level (less than high school, high school/GED, or above high school), marital status, family PIR [income poverty ratio, categorized as low income (< 1.3), moderate-income (1.3–3.49), or high income (≥ 3.5)]; (b) Lifestyle behaviors, including drinking (categorized as yes or no by more or less than 12 drinks per year) and smoking status (categorized as yes or no by more or less than 100 cigarettes in lifetime); (c) Clinical characteristics, including body mass index (BMI, kg/m^2^), menopause, diabetes, hypertension, dyslipidemia, and cardiovascular disease (each indicated as yes or no). Comprehensive data on these variables are accessible through the public database of the NHANES (https://www.cdc.gov/nchs/nhanes/).

### Statistical analysis

The statistical examination of the data was carried out using R software (version 4.3.2) and EmpowerStats (version 4.2). Differences in baseline characteristics between groups were analyzed using one-way ANOVA tests (normally distributed continuous variables), Student’s t-tests (non-normally distributed continuous variables), and chi-square tests (categorical variables). Weighted multivariate linear regression analyses and logistic regression analyses were used to determine the relationship between pregnancy loss and depressive symptoms. Model 1adjusted for age, race, education level, marital status and family PIR; Model 2 adjusted for age, race, education level, marital status, family PIR, age at menarche, age at first delivery, age at last delivery, number of vaginal deliveries, and number of cesarean deliveries; and Model 3 made comprehensive adjustments for all covariates, including Model 2, drinking, smoking, BMI, menopause, diabetes, hypertension, dyslipidemia, and CVD. Smoothed curve fitting and generalized additive models were used to explore nonlinear relationships between pregnancy loss and depression. To assess the stability of the findings, we performed sensitivity analyses. First, multivariate linear regression analyses were performed with PHQ-9 scores as continuous variables. Second, multivariate logistic regression analyses were performed at a critical PHQ-9 score of 5. In addition, we conducted subgroup analyses to explore whether there were other confounding factors, such as age, race, education level, marital status, and family PIR, that might influence the relationship between pregnancy loss and depression.

## Results

### Baseline characteristics

Our analysis included 12,873 female participants enrolled between 2007 and 2020, characterized by an average age of 53.35 ± 16.11 years. Table 1 shows the baseline characteristics of individuals based on depressive symptoms: out of the total sample size, a total of 1,595 individuals reported having depressive symptoms (PHQ-9 ≥ 10), representing 12.39% of the study population. Female participants with higher PHQ-9 scores were more likely to have a lower level of education, lower income, be more likely to be current smokers and consume alcohol, be more likely to have a higher Body Mass Index (BMI), and have diabetes mellitus, hypertension, hyperlipidemia, and cardiovascular disease. In addition, race and marital status were associated with depressive symptoms, and being unmarried, widowed, divorced, and spousal separation were also associated with having a higher PHQ-9 score. Women with depressive symptoms had younger age, age at menarche, age at first delivery, and age at last delivery than women without depressive symptoms.

**Table 1:** Baseline characteristics of study population

Variable	Total	Non-depression	Depression	*p*-value
**Demographic and socioeconomic information**				
Age (years)	53.35 ± 16.11	53.61 ± 16.22	51.55 ± 15.16	< 0.001
Age at menarche (years)	12.74 ± 1.80	12.78 ± 1.78	12.49 ± 1.91	< 0.001
Age at first delivery (years)	21.83 ± 4.27	22.00 ± 4.31	20.60 ± 3.76	< 0.001
Age at last delivery (years)	28.49 ± 5.72	28.61 ± 5.72	27.65 ± 5.64	< 0.001
Number of pregnancies	3.54 ± 2.02	3.47 ± 1.95	4.00 ± 2.43	< 0.001
Number of live births	2.74 ± 1.57	2.72 ± 1.55	2.90 ± 1.74	< 0.001
Number of vaginal deliveries	1.81 ± 1.91	1.80 ± 1.89	1.93 ± 2.05	0.088
Number of cesarean deliveries	0.92 ± 1.31	0.92 ± 1.30	0.97 ± 1.39	0.305
Race (%)				< 0.001
Mexican American	1973 (15.33)	1713 (15.19)	260 (16.30)	
Other Hispanic	1520 (11.81)	1263 (11.20)	257 (16.11)	
Non-Hispanic White	5161 (40.09)	4543 (40.28)	618 (38.75)	
Non-Hispanic Black	2951 (22.92)	2597 (23.03)	354 (22.19)	
Other Races	1268 (9.85)	1162 (10.30)	106 (6.65)	
Education level (%)				< 0.001
Less than high school	3314 (25.74)	2724 (24.15)	590 (36.99)	
High school or GED	3026 (23.51)	2646 (23.46)	380 (23.82)	
Above high school	6533 (50.75)	5908 (52.39)	625 (39.18)	
Marital (%)				< 0.001
Married/ Living with a partner	7283 (56.58)	6597 (58.49)	686 (43.01)	
Widowed/ Divorced/ Separated	4297 (33.38)	3613 (32.04)	684 (42.88)	
Never married	1293 (10.04)	1068 (9.47)	225 (14.11)	
Family PIR (%)				< 0.001
< 1.31	4081 (31.70)	3278 (29.07)	803 (50.34)	
1.31–3.49	5657 (43.94)	5036 (44.65)	621 (38.93)	
≥ 3.50	3135 (24.35)	2964 (26.28)	171 (10.72)	
**Lifestyle behaviors**				
Drinking (%)				0.002
No	6093 (47.33)	5397 (47.85)	696 (43.64)	
Yes	6780 (52.67)	5881 (52.15)	899 (56.36)	
Smoking (%)				< 0.001
No	8007 (62.20)	7291 (64.65)	716 (44.89)	
Yes	4866 (37.80)	3987 (35.35)	879 (55.11)	
**Clinical characteristics**				
BMI (kg/m^2^)	30.28 ± 7.58	30.03 ± 7.40	32.03 ± 8.55	< 0.001
Menopause (%)				0.549
No	9460 (73.49)	8278 (73.40)	1182 (74.11)	
Yes	3413 (26.51)	3000 (26.60)	413 (25.89)	
Diabetes (%)				< 0.001
No	10,485 (81.45)	9310 (82.55)	1175 (73.67)	
Yes	2388 (18.55)	1968 (17.45)	420 (26.33)	
Hypertension (%)				< 0.001
No	7071 (54.93)	6298 (55.84)	773 (48.46)	
Yes	5802 (45.07)	4980 (44.16)	822 (51.54)	
Dyslipidemia (%)				< 0.001
No	3362 (26.12)	3050 (27.04)	312 (19.56)	
Yes	9511 (73.88)	8228 (72.96)	1283 (80.44)	
CVD (%)				< 0.001
No	11,850 (92.05)	10,493 (93.04)	1357 (85.08)	
Yes	1023 (7.95)	785 (6.96)	238 (14.92)	

Abbreviations: BMI, body mass index; Family PIR, family poverty income ratio; CVD, cardiovascular disease

### Results of unadjusted logistic regression model

To further examine the association between each of the participants’ characteristics and depressive symptoms, we used univariate logistic analysis. The results showed that age, age at menarche, age at first delivery, age at last delivery, number of pregnancies, number of live births, number of vaginal deliveries, education level, marital status, family PIR, alcohol consumption status, smoking status, body mass index, diabetes mellitus, hypertension, hyperlipidemia, and cardiovascular disease were significantly associated with depression (*p* < 0.05, Table 2).

**Table 2:** Association of covariates and depression risk

Character	OR (95% CI)	*p*
**Demographic and socioeconomic information**		
Age (years)	0.99 (0.99, 1.00)	< 0.0001
Age at menarche (years)	0.91 (0.89, 0.94)	< 0.0001
Age at first delivery (years)	0.91 (0.90, 0.93)	< 0.0001
Age at last delivery (years)	0.97 (0.96, 0.98)	< 0.0001
Number of pregnancies	1.12 (1.10, 1.15)	< 0.0001
Number of live births	1.07 (1.04, 1.11)	< 0.0001
Number of vaginal deliveries	1.03 (1.01, 1.06)	0.0126
Number of cesarean deliveries	1.03 (0.99, 1.08)	0.0888
Race		
Non-Hispanic White	Reference	
Non-Hispanic Black	1.00 (0.87, 1.15)	0.9771
Mexican American	1.12 (0.96, 1.30)	0.1665
Other Hispanic	1.50 (1.28, 1.75)	< 0.0001
Other Races	0.67 (0.54, 0.83)	0.0003
Education level		
Less than high school	Reference	
High school or GED	0.66 (0.58, 0.76)	< 0.0001
Above high school	0.49 (0.43, 0.55)	< 0.0001
Marital		
Married/ Living with a partner	Reference	
Widowed/ Divorced/ Separated	1.82 (1.63, 2.04)	< 0.0001
Never married	2.03 (1.72, 2.39)	< 0.0001
Family PIR		
<1.31	Reference	
1.31–3.49	0.50 (0.45, 0.56)	< 0.0001
≥3.50	0.24 (0.20, 0.28)	< 0.0001
**Lifestyle behaviors**		
Drinking		
No	Reference	
Yes	1.19 (1.07, 1.32)	0.0016
Smoking		
No	Reference	
Yes	2.25 (2.02, 2.50)	< 0.0001
**Clinical characteristics**		
BMI (kg/m2)	1.03 (1.03, 1.04)	< 0.0001
Menopause		
No	Reference	
Yes	0.96 (0.86, 1.09)	0.5493
Diabetes		
No	Reference	
Yes	1.69 (1.50, 1.91)	< 0.0001
Hypertension		
No	Reference	
Yes	1.34 (1.21, 1.49)	< 0.0001
Dyslipidemia		
No	Reference	
Yes	1.52 (1.34, 1.74)	< 0.0001
CVD		
No	Reference	
Yes	2.34 (2.01, 2.74)	< 0.0001

### Results of adjusted logistic regression model

We created four models to assess the independent effects of pregnancy loss on depression. Table 3 shows the effect sizes, ORs, and 95% CIs derived from the multivariate logistic regression models. In the unadjusted model, women with one pregnancy loss had a 32% increased risk of depression compared with women with no experience of pregnancy loss (OR = 1.32; 95% CI 1.16, 1.50). After adjusting for confounders, the OR was 1.31 (95% CI 1.15, 1.50) (*p* < 0.0001). Similarly, in the unadjusted model, women who had two or more pregnancy losses had an 80% increased risk of depression compared with women who had no experience of pregnancy loss (OR = 1.80; 95% CI 1.59, 2.05). After adjusting for confounders, the OR was 1.58 (95% CI 1.38, 1.81) (*p* < 0.0001). This significant OR suggests that the number of pregnancy losses is a risk factor for the development of depressive symptoms.

**Table 3:** Multivariable-adjust ORs and 95%CI of pregnancy loss associated with depression

Number of pregnancy losses	Non-Adjusted Model		Model 1		Model 2		Model 3	
	OR (95%CI)	*p*-value	OR (95%CI)	*p*-value	OR (95%CI)	*p*-value	OR (95%CI)	*p*-value
0	Reference		Reference		Reference		Reference	
1	1.32 (1.16, 1.50)	< 0.0001	1.35 (1.19, 1.54)	< 0.0001	1.34 (1.17, 1.53)	< 0.0001	1.31 (1.15, 1.50)	< 0.0001
2+	1.80 (1.59, 2.05)	< 0.0001	1.74 (1.52, 1.98)	< 0.0001	1.71 (1.50, 1.96)	< 0.0001	1.58 (1.38, 1.81)	< 0.0001

Model 1: adjusted for age, race, education, marital status, and family PIR.

Model 2: adjusted for Model 1, age at menarche, age at first delivery, age at last delivery, number of vaginal deliveries, and number of cesarean deliveries.

Model 3: adjusted for Model 2, drinking, smoking, BMI, menopause, diabetes, hypertension, dyslipidemia, and CVD

**Fig. 2:**
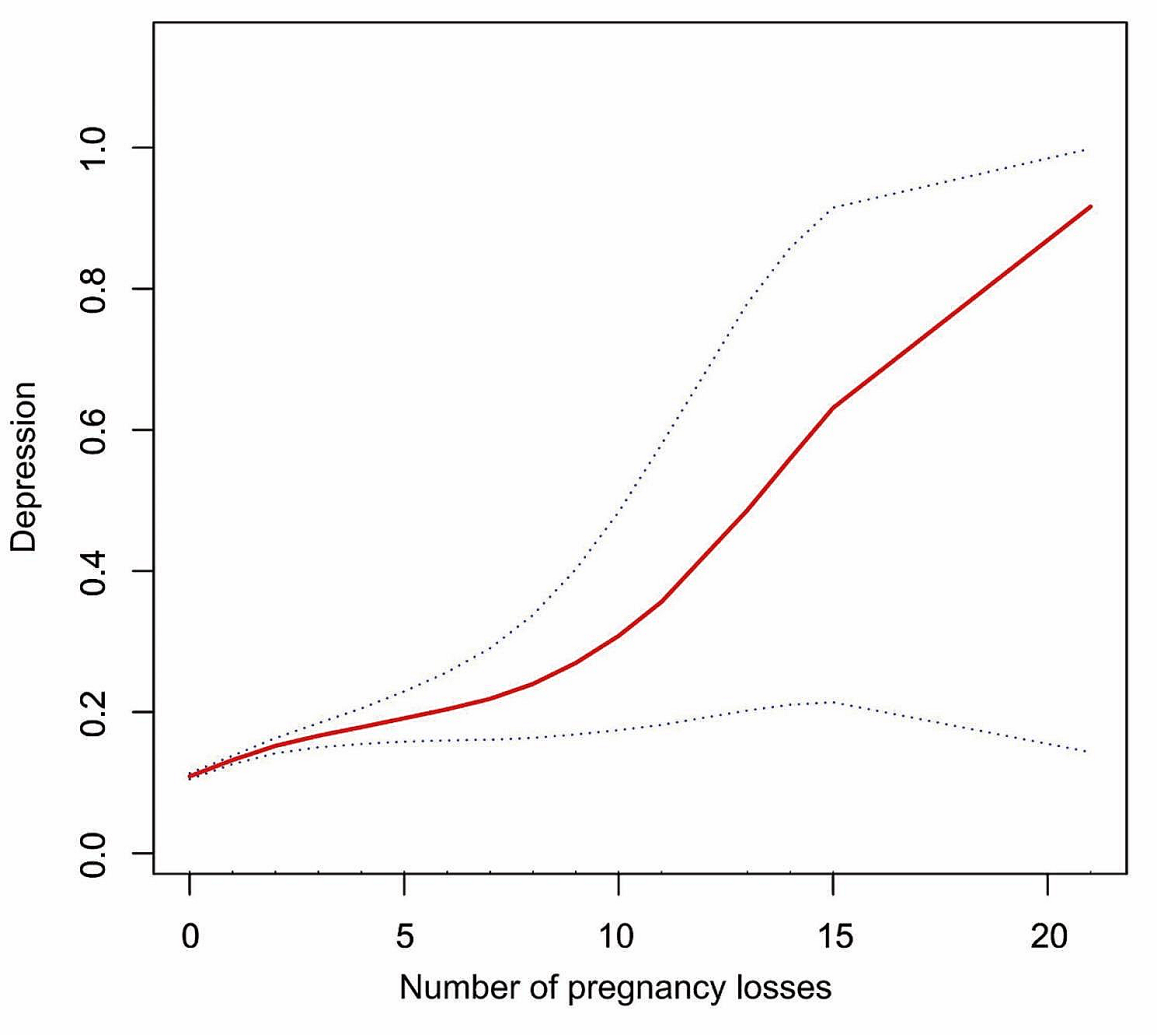
Association between pregnancy loss and depression

Moreover, the positive correlation between pregnancy loss and depressive symptoms was further validated by the results of the smoothed-fitted curves (Fig. 2). As the number of pregnancy losses increased, there was a gradual increase in reporting the occurrence of depressive symptoms.

### Sensitivity analyses

Table 4 summarizes the results of the sensitivity analyses. When linear regression was performed with the PHQ-9 score as a continuous variable, the linear regression results in all models were statistically significant (*p* < 0.0001), indicating that the number of pregnancy losses was positively correlated with the continuous PHQ-9 score (for 1 loss: β = 0.60, 95% CI 0.41,0.79; for 2 or more losses: β = 1.03, 95% CI 0.82, 1.24). When PHQ-9 scores were used as a categorical variable in logistic regression, with a threshold of 5, the results similarly indicated that pregnancy loss was associated with a high risk of depression (for 1 loss: OR = 1.25, 95% CI 1.14,1.37; for 2 or more losses: OR = 1.48, 95% CI 1.34,1.64).

**Table 4:** Sensitivity analyses

Analyses	Non-Adjusted Model		Model 1		Model 2		Model 3	
linear regression								
	β (95%CI)	*p*-value	β (95%CI)	*p*-value	β (95%CI)	*p*-value	β (95%CI)	*p*-value
0	Reference		Reference		Reference		Reference	
1	0.64 (0.44, 0.84)	< 0.0001	0.67 (0.47, 0.86)	< 0.0001	0.66 (0.47, 0.85)	< 0.0001	0.60 (0.41, 0.79)	< 0.0001
2+	1.34 (1.12, 1.55)	< 0.0001	1.22 (1.01, 1.43)	< 0.0001	1.22 (1.00, 1.43)	< 0.0001	1.03 (0.82, 1.24)	< 0.0001
**logistic regression (PHQ-9 score cut-off = 5)**								
	**OR (95%CI)**	***p*** **-value**	**OR (95%CI)**	***p*** **-value**	**OR (95%CI)**	***p*** **-value**	**OR (95%CI)**	***p*** **-value**
0	Reference		Reference		Reference		Reference	
1	1.24 (1.14, 1.36)	< 0.0001	1.28 (1.16, 1.40)	< 0.0001	1.28 (1.16, 1.40)	< 0.0001	1.25 (1.14, 1.37)	< 0.0001
2+	1.62 (1.47, 1.78)	< 0.0001	1.58 (1.43, 1.74)	< 0.0001	1.58 (1.43, 1.75)	< 0.0001	1.48 (1.34, 1.64)	< 0.0001

Model 1: adjusted for age, race, education, marital status, and family PIR.

Model 2: adjusted for Model 1, age at menarche, age at first delivery, age at last delivery, number of vaginal deliveries, and number of cesarean deliveries.

Model 3: adjusted for Model 2, BMI, drinking, smoking, menopause, diabetes, hypertension, dyslipidemia, and CVD

### Subgroup analyses

To examine potential differences in the relationship between pregnancy loss and depression in specific populations, we conducted subgroup analyses and interaction tests by age, race, education level, marital status, and family PIR. Table 5 shows that the positive association between pregnancy loss and depressive symptoms remained significant across subgroups, including different age, education level, and marital status groups. However, the association between pregnancy loss and depression was not statistically significant in high-income populations (family PIR ≥ 3.50). Also, in the adjusted model, we observed a significant interaction between family PIR and depression (*p* for interaction < 0.05).

**Table 5:** Subgroup analysis of the association between pregnancy loss and depression

Subgroup	OR (95%CI)	*p* for interaction
Age (years)		0.1083
< 50	1.12 (1.06, 1.19)	
≥ 50	1.20 (1.13, 1.26)	
Race		0.1032
Mexican American	1.21 (1.09, 1.34)	
Other Hispanic	1.13 (1.02, 1.25)	
Non-Hispanic White	1.21 (1.14, 1.30)	
Non-Hispanic Black	1.11 (1.03, 1.21)	
Other races	1.03 (0.91, 1.16)	
Education level		0.3187
Less than high school	1.19 (1.11, 1.27)	
High school or GED	1.10 (1.01, 1.19)	
Above high school	1.17 (1.10, 1.24)	
Marital		0.8803
Married/ Living with a partner	1.17 (1.10, 1.23)	
Widowed/ Divorced/ Separated	1.15 (1.08, 1.22)	
Never married	1.18 (1.06, 1.31)	
Family PIR		0.0293
< 1.31	1.18 (1.12, 1.25)	
1.31–3.49	1.16 (1.09, 1.24)	
≥ 3.50	0.95 (0.81,1.12)	

Adjusted for all covariates except effect modifier

## Discussion

This is a cross-sectional study investigating the relationship between pregnancy loss and depressive symptoms among adult women in the United States. The findings underscored a significant correlation between pregnancy loss and depression, holding even when adjustments were made for various covariates. These results align with our preliminary hypothesis, showing that experiencing pregnancy loss is associated with depressive symptoms in women. It is important to emphasize that as the number of pregnancy loss events increased, women were also at significantly increased risk for depression. Given these insights, we advocate for the inclusion of adverse pregnancy event documentation as part of a comprehensive approach to managing depression in women.

Supporting our conclusions, prior research has similarly indicated a connection between pregnancy loss and depressive symptoms, highlighting the complex dynamics of this relationship. A systematic review pointed to the detrimental effects of pregnancy complications on mental health, emphasizing that such adverse events significantly raise the risk of depressive symptoms [19]. Furthermore, research incorporating both qualitative interviews and quantitative analysis revealed that women who have undergone one or more abortions face a markedly higher risk of postpartum depression and post-traumatic stress compared to those who haven’t experienced such losses [20, 21]. This is corroborated by findings from Westby [22] and Arocha [23], who noted the long-lasting negative impact of stillbirth on women’s mental health, particularly increasing the likelihood of depression and anxiety. In addition, large differences in the meantime since the adverse pregnancy event were observed in cross-sectional studies of the same type [24, 25]. These studies demonstrate the long-term mental health disorders that experiencing pregnancy loss can have on women. However, not all studies support a direct association between pregnancy loss and depression. A quantitative study in Australia failed to find a significant correlation between abortion experience and depressive symptoms. The researchers noted that adapting to a miscarriage is often emotionally challenging, but is not always associated with poor mental health, which may be related to an individual’s psychological resilience, the strength of their social support system, and the impact of other life events [26]. This suggests that we need to consider broader psychosocial factors when assessing the relationship between these pregnancy experiences and mental health status. Differences in the results of these studies may stem from a variety of factors, including differences in study design, diversity in data collection methods, and differences in participant demographic characteristics. Despite individual contrary findings, a large body of evidence suggests a complex association between pregnancy loss and depression.

The etiologic relationship between pregnancy loss and depression remains an under-addressed question within the field of medical research. Although current data are not sufficient to support direct causal inferences, the research findings that do exist provide possible pathways to a deeper understanding of this complex relationship. The dramatic shifts in hormone levels (including steroid and peptide hormones) during pregnancy, and their abrupt alteration due to adverse pregnancy events, can disrupt neurotransmitter balance in the brain, potentially leading to depressive moods [27]. Furthermore, miscarriages and stillbirths can initiate an inflammatory response in the body, aligning with the biological mechanisms associated with depression, such as the elevation of pro-inflammatory cytokine levels, including high-sensitivity C-reactive protein, thereby impacting mood regulation and brain function [28, 29]. Psychosocial dynamics also significantly contribute to the connection between pregnancy loss and depression. The profound emotional turmoil, sense of loss, and societal perceptions of these events can severely affect mental well-being [30]. The diminishment of social support and empathy can amplify feelings of isolation, whereas self-directed blame, guilt, or apprehension about the future may culminate in heightened psychological stress, elevating depression risk. Moreover, the disruption of personal life plans and expectations due to fertility challenges can lead to intense psychological and emotional distress [31, 32]. The economic ramifications of pregnancy loss, particularly on families with limited financial resources, encompassing healthcare costs and income loss during recuperation, can intensify existing psychological pressures [33]. Additionally, the interplay between genetic predispositions and environmental stressors, including pregnancy loss, can activate an individual’s vulnerability to depression, facilitating the onset of depressive episodes [34]. In essence, the association between pregnancy loss and depression in women may be due to a combination of hormonal fluctuations, inflammatory responses, psychosocial pressures, financial impacts, and genetic vulnerabilities. A comprehensive understanding of these factors is essential for devising effective preventative measures and treatment modalities, alongside furnishing adequate support and intervention for women navigating these challenging experiences.

The results of the subgroup analyses showed that the association between pregnancy loss and depression exhibited variability across subgroups with different levels of household income. It may be the case that the family’s economic situation moderates the association between pregnancy loss and depression in specific populations [35]. Higher-income populations may have lower socioeconomic stress, more specialized mental health care resources [36], and other factors that may have reduced the association between pregnancy loss and depression. The presence of this moderating effect suggests that individuals from different economic backgrounds may have different coping capacities or risks for psychological reactions related to pregnancy loss. We, therefore, recommend that in public health and clinical practice, the different risks and needs that individuals from different economic backgrounds may have are considered to provide more effective support and interventions [37].

The strength of our study lies in utilizing the National Health and Nutrition Examination Survey (NHANES) data, providing a comprehensive and representative sample through detailed sampling methods. By adjusting for potential lifestyle and clinical confounders, we’ve bolstered the credibility of our findings. We also conducted subgroup analyses to investigate the robustness of the relationship between pregnancy loss and depression in different populations. Despite the keen interest in the connection between pregnancy loss and depression, research faces hurdles such as our study’s inability to establish causality due to its cross-sectional design. Depression may have already been higher in the pregnancy loss group before their pregnancies and pregnancy loss is instead a mediator. In addition, the data set does not contain information on different forms of pregnancy losses and neither does it include time since miscarriages, and so the association between different forms of pregnancy losses/time since miscarriage and depression is not possible to assess. Based on previous research [38], there is a potential for missing not at random (MNAR) regarding the missing exposure and outcome data in this study. Because of the sensitivity of these questions or the different questionnaire fatigue in severely depressed individuals, the possibility exists that participants selectively avoided answering these questions. Additionally, while tools like the PHQ-9 scale are useful for assessing depressive symptoms, they cannot replace clinical diagnoses and may be influenced by personal biases and emotional states at the time of completion.

## Conclusion

Our study suggests that pregnancy loss was associated with elevated PHQ-9 scores and an increased likelihood of depression. This study may provide a basis for strategies to mitigate the impact of these adverse events on women’s mental health.

## Data Availability

This study made use of publicly available datasets, with all the data pertinent to our research accessible via the official National Health and Nutrition Examination Survey website: https://www.cdc.gov/nchs/nhanes/.
